# Modelling optimal use of temporarily restricted colonoscopy capacity in a FIT-based CRC screening program: Application during the COVID-19 pandemic

**DOI:** 10.1371/journal.pone.0270223

**Published:** 2022-06-24

**Authors:** Lucie de Jonge, Hilliene J. van de Schootbrugge-Vandermeer, Emilie C. H. Breekveldt, Manon C. W. Spaander, Hanneke J. van Vuuren, Folkert J. van Kemenade, Evelien Dekker, Iris D. Nagtegaal, Monique E. van Leerdam, Iris Lansdorp-Vogelaar

**Affiliations:** 1 Department of Public Health, Erasmus MC University Medical Center, Rotterdam, The Netherlands; 2 Department of Gastroenterology and Hepatology, Netherlands Cancer Institute – Antoni van Leeuwenhoek Hospital, Amsterdam, The Netherlands; 3 Department of Gastroenterology and Hepatology, Erasmus MC University Medical Center, Rotterdam, The Netherlands; 4 Department of Pathology, Erasmus MC University Medical Center, Rotterdam, The Netherlands; 5 Department of Gastroenterology and Hepatology, Amsterdam University Medical Centers, Location AMC, Amsterdam, The Netherlands; 6 Department of Pathology, Radboud University Medical Centre, Nijmegen, The Netherlands; 7 Department of Gastroenterology and Hepatology, Leiden University Medical Center, Leiden, the Netherlands; University of Oslo, NORWAY

## Abstract

**Objective:**

The COVID-19 pandemic forced colorectal cancer (CRC) screening programs to downscale their colonoscopy capacity. In this study, we assessed strategies to deal with temporary restricted colonoscopy capacity in a FIT-based CRC screening program while aiming to retain the maximum possible preventive effect of the screening program.

**Design:**

We simulated the Dutch national CRC screening program inviting individuals between ages 55 and 75 for biennial FIT using the MISCAN-Colon model including the 3-month disruption in the first half of 2020 due to the COVID-19 pandemic. For the second half of 2020 and 2021, we simulated three different strategies for the total target population: 1) increasing the FIT cut-off, 2) skipping one screening for specific screening ages, and 3) extending the screening interval. We estimated the impact on required colonoscopy capacity in 2020–2021 and life years (LYs) lost in the long-term.

**Results:**

Increasing the FIT cut-off, skipping screening ages and extending the screening interval resulted in a maximum reduction of 25,100 (-17.0%), 16,100(-10.9%) and 19,000 (-12.9%) colonoscopies, respectively. Modelling an increased FIT cut-off, the number of LYs lost ranged between 1,400 and 4,400. Skipping just a single screening age resulted in approximately 2,700 LYs lost and this was doubled in case of skipping two screening ages. Extending the screening interval up to 34 months had the smallest impact on LYs lost (up to 1,100 LYs lost).

**Conclusion:**

This modelling study shows that to anticipate on restricted colonoscopy capacity, temporarily extending the screening interval retains the maximum possible preventive effect of the CRC screening program.

## Introduction

The world has been dealing with a sudden pandemic due to the COVID-19 virus since 2020. During the first wave, non-acute health care was down-scaled. Many health services (i.e. disease prevention, diagnosis and treatment) were postponed due to capacity problems. This was due to for example lacking protective cloths, health care workers being appointed at COVID-19 units in hospitals and possible risk of getting infected. Consequently, many colorectal cancer (CRC) screening programs world-wide were disrupted.

Modelling studies have estimated that disruptions to faecal immunochemical testing (FIT)-based CRC screening programs could negatively impact CRC incidence and CRC-related mortality in the long-term [1, 2]. Therefore, it is important to keep disruptions of screening programs as short as possible and after restart encourage individuals to participate. Moreover, catching up individuals’ missed invitations is pivotal to reduce the long-term impact on outcomes of CRC screening.

Due to decreasing numbers of COVID-19 cases, many CRC screening programs across the world were able to restart again, albeit often at restricted capacity, if possible starting with catching up the backlog. However, subsequent waves of the pandemic have been occurring and are still expected. Health services are still under pressure and are forced to downscale, including colonoscopy capacity in CRC screening programs. As a consequence, many programs currently do not have capacity to screen their entire target population, let alone catch-up the backlog of individuals missed during the disruption in the first wave of the pandemic.

There is still uncertainty, however, on the best strategy to restart the screening with restricted colonoscopy capacity. In the past, microsimulation models were successfully used to inform a FIT-based CRC screening program on the most appropriate strategy in case of restricted colonoscopy capacity [3]. However, this previous study did not assess temporarily strategies and it is therefore unknown how to best deal with only a temporary shortages in colonoscopy capacity.

Considering the special circumstances and temporarily restricted colonoscopy capacity, we aimed to determine the best strategy to deal with these shortages in capacity using the MIcrosimulation SCreening ANalysis for CRC model (MISCAN-Colon). We compared the effect of temporarily increasing the FIT cut-off, skipping certain age-groups and extending the screening interval on short-term outcomes (i.e. colonoscopy demand) and long-term outcomes (CRC incidence, mortality and LYs lost) of CRC screening programs.

## Materials and methods

We used the MISCAN-Colon model to simulate the Dutch national CRC screening program under different screening strategies to anticipate on temporary restricted colonoscopy capacity. We compared the effect of these strategies on short-term reductions in capacity and long-term CRC incidence and mortality to determine which strategy provided the best balance between colonoscopy capacity and screening benefit.

### National FIT-based CRC screening program in the Netherlands

The Dutch national FIT-based CRC screening program invites individuals between ages 55 and 75 years every two years to perform a FIT. The FIT positivity cut-off is set to 47 μg Hb/g faeces. The program was implemented gradually by age cohort from 2014 onwards, with a roll-out period of five years, eventually being offered to a target population of approximately 2.2 million [4]. In case of a positive FIT, individuals are referred for a follow-up colonoscopy. Participants are referred for further treatment and surveillance according to the Dutch surveillance guidelines [5]. Due to the COVID-19 pandemic, the Dutch national CRC screening program was disrupted from March 16 to May 11, 2020 [6]. In June 2020, the program restarted at a moment that only 35% of the original colonoscopy capacity was available. By September 2020, the available colonoscopy capacity was back at its original level [7].

### MISCAN-Colon

The MISCAN-Colon model is a well-established microsimulation model for CRC developed at the Department of Public Health, Erasmus MC University Medical Center and has been extensively described previously [3, 8]. In brief, the model simulates the life-histories of a large population of individuals from birth to death. In addition, the model simulates the development of CRC through the adenoma-carcinoma sequence. As each simulated individual ages, one or more adenomas may develop and these adenomas can progress in size from small (≤ 5 mm) to medium (6–9 mm) to large (≥10 mm). Some adenomas can develop into preclinical cancer, which may progress through cancer stages I to IV. At any time during the development of the disease, symptoms may present and CRC may be diagnosed. By introducing screening, the simulated life-histories may be altered through detection and removal of adenomas or CRC at an earlier stage with a more favourable prognosis. By comparing the life-histories of a simulated population undergoing screening to the corresponding life-histories in a simulated population without screening, MISCAN-Colon is able to quantify the effectiveness and costs of screening.

In this study, MISCAN-Colon simulated the Dutch population with CRC screening program using a sample size of 500 million individuals to eliminate the effect of randomness in the model, taking the gradual roll-out from 2014 until 2019 into account. The model for the Dutch population was calibrated to data on age-, stage- and location-specific CRC incidence obtained from the Netherlands Cancer Registry and age-specific prevalence and multiplicity distribution of adenomas from autopsy and colonoscopy studies [9–19]. Age-, and invitation round-specific FIT and colonoscopy participation rates were based on data from the Dutch national CRC screening program provided by the Dutch national screening database [20]. Also, we modelled the disruption of the national CRC screening program of 3 months in 2020 due to the COVID-19 pandemic.

### Modelled strategies

First, we simulated an ideal scenario in which invitations for individuals due for screening during the disruption period, were caught up during the first three months after the restart of the program (comparator strategy, S1 Fig in S1 File).

Next, we considered three types of strategies to temporarily reduce required colonoscopies in the program in order to meet reduced capacity due to the COVID-19 pandemic:

1. Increasing the cut-off value of the FIT from 47 μg Hb/g faeces to (S2 Fig in S1 File)
  - 50 μg Hb/g faeces,
  - 55 μg Hb/g faeces,
  - 60 μg Hb/g faeces, or
  - 70 μg Hb/g faeces.
2. Skipping specific screening age for one screening (S2 Fig in S1 File)
  - 55-year-old individuals,
  - 63-year-old individuals, or
  - 63- and 65-year-old individuals.
3. Extending the FIT screening interval from 24 months to (S3 Fig in S1 File)
  - 28 months,
  - 30 months,
  - 32 months,
  - 34 months, or
  - 36 months.

For first-time invitees, the invitation was postponed equal to the extension for subsequent round invitees (i.e. extending the screening interval to 28 months resulted in a delay for first-time invitees of 4 months). The age group 55-year-old individuals was chosen for skipping one screening, because this is the group with the lowest CRC risk. Age groups 63-year-old individuals and 65-year-old individuals were chosen, because these were the youngest age groups that already received two invitations for the Dutch CRC screening, and therefore considered as having a lower CRC risk compared other age groups in the program. In our model, these strategies were temporarily applied for 18 months after restart of the program in July 2020. After 18 months, it was assumed that program performance was back to normal.

### Test characteristics

Test characteristics for all FIT cut-offs were estimated so that the model predicted positivity and detection rates for advanced adenomas and CRC was similar to those observed in the CRC screening program in 2014 and 2015 [21]. Since the data contained information about haemoglobin concentrations and detection of all individuals with a positive FIT using a cut-off of 47 μg Hb/g faeces, we were able to estimate positivity- and detection rates for higher cut-offs. Test characteristics for follow-up and surveillance colonoscopy were based on a systematic review of polyp miss rates in tandem colonoscopy studies [22]. Test characteristics, positivity- and detection rates for all FITs can be found in S1 Table (S1 File).

### Outcomes

In this study, short-term outcomes of interest were the cumulatively colonoscopy demand in 2020 and 2021 and yearly between 2020 and 2024. Long-term outcomes of interest were cumulative CRC incidence, CRC-related mortality and life years (LYs) lost between 2020 and 2050. Outcomes for the comparator strategy were given in absolute numbers while taking into account the population size. The outcomes for each alternative strategy were presented as absolute and relative changes relative to the comparator strategy. All strategies were evaluated in efficiency measures calculated by increase in CRC incidence, CRC-related deaths and LYs lost per colonoscopy not performed in 2020 and 2021 compared to the comparator strategy. For the best strategy based on the efficiency measures, change in CRC incidence by stage between 2020 and 2023 was given compared to the comparator strategy.

### Sensitivity analyses

We conducted three sensitivity analyses. First, in line with previous literature [23, 24], we assumed a 10 percentage point lower participation to FIT screening during the second half of 2020 and the full year of 2021 for all strategies (i.e. in the Netherlands, overall FIT participation rate is ±73%, and now assumed to be 63%). Second, in case of temporarily extended screening interval, we assumed that individuals would receive one fewer invitation if the simulated age in our model of their last invitation would exceed age 75 years, the current stopping age for the Dutch CRC screening program (i.e. individuals would receive 10 invitations instead of 11 invitations). Last, we assumed a restriction period of 24 months (i.e. during the second half of 2020, the full year of 2021 and the first half of 2022) in our base case strategies.

## Results

### Comparator strategy

**Fig 1:**
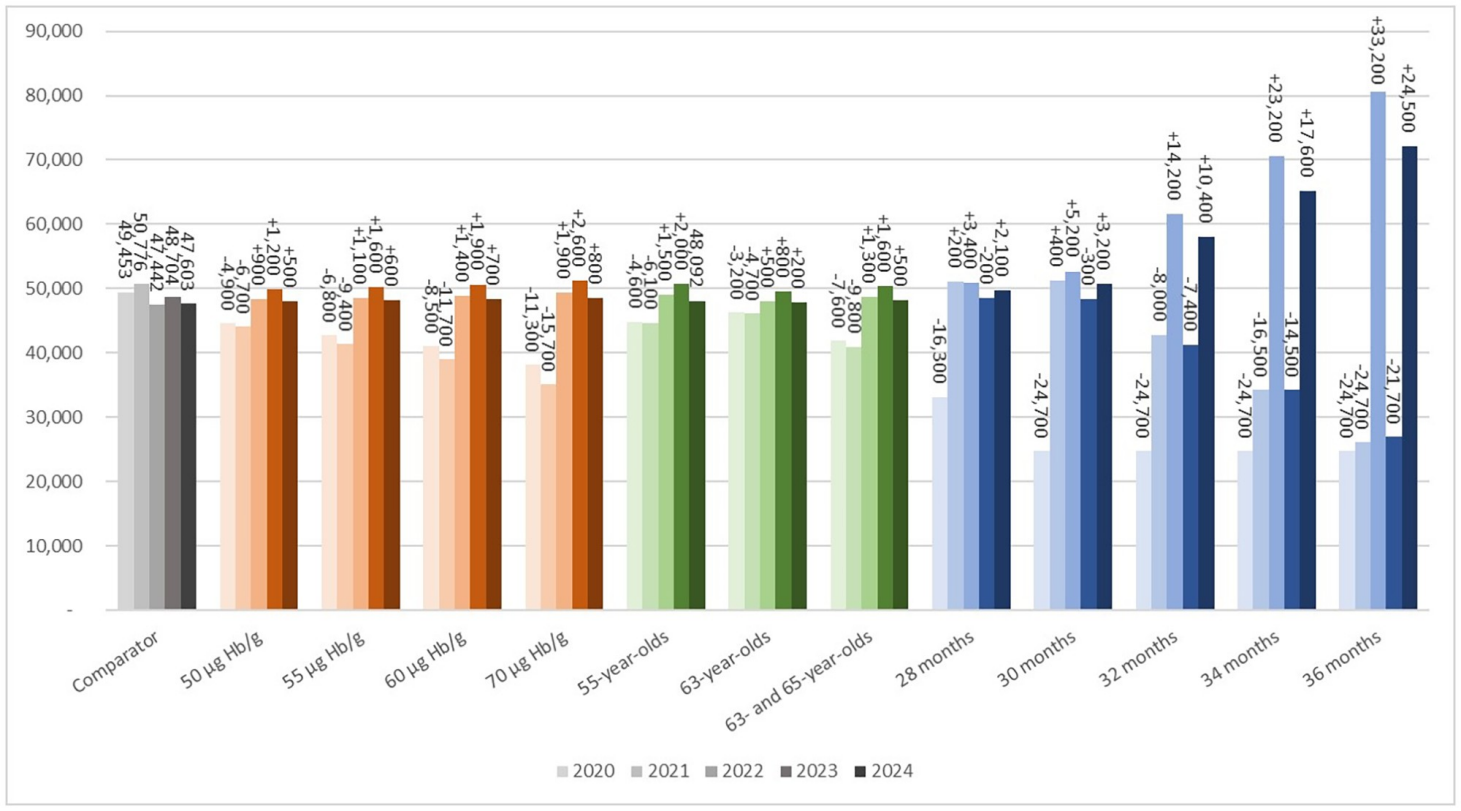
Colonoscopy demand for the comparator strategy, increasing the cut-off value, skipping screening ages and extending the screening interval for 2020, 2021, 2022, 2023 and 2024. Note that in the comparator scenario, we simulated the Dutch CRC screening program including the 3-month disruption from April 2020 and after the disruption individuals’ missed invitations were caught up in the next 3 months. Abbreviations: CRC, colorectal cancer; μg Hb/g, microgram Haemoglobin per gram.

In the absence of any restriction in colonoscopy capacity in the post-disruption period (comparator strategy), approximately 148,000 follow-up colonoscopies were estimated to be required after a positive FIT in 2020,2021, and 2022 (Fig 1). From 2020–2050, cumulatively 173,800 CRC-related deaths and 554,100 LYs were estimated.

### Increasing FIT cut-off

Increasing the FIT cut-off in the second half of 2020 and in the full year of 2021 from 47 to 50, 55, 60 and 70 μg Hb/g faeces resulted in an estimated reduction in colonoscopy demand of 11,700 (7.3%), 15,000 (10.2%), 18,700 (12.7%) and 25,100 (17.0%), respectively, in 2020,2021, and 2022 (Table 1). When the FIT cut-off was temporarily increased to 50, 55, 60 and 70 μg Hb/g faeces, respectively 400, 600, 700 and 900 (0.08%, 0.12%, 0.14%, 0.18%) more individuals would have died as a result of CRC from 2020 to 2050. The number of LYs lost were estimated to increase by up to 4,400 (0.80%).

**Table 1:** The efficiency of strategies to reduce colonoscopy demand predicted by MISCAN-Colon.

	Reduction in colonoscopy demand in 2020, 2021 and 2022 (%)	Excess CRC incidence (2020–2050, %)	Increase in CRC incidence per colonoscopy not performed	Excess CRC deaths (2020–2050, %)	Increase in CRC deaths per colonoscopy not performed	Excess LYs lost (2020–2050, %)	Increase in LYs lost per colonoscopy not performed
*Increasing the cut-off value*							
50 μg Hb/g faeces	11,700 (7.3%)	400 (0.08%)	0.04	200 (0.09%)	0.01	1,400 (0.25%)	0.18
55 μg Hb/g faeces	15,000 (10.2%)	600 (0.12%)	0.04	300 (0.15%)	0.02	2,400 (0.43%)	0.15
60 μg Hb/g faeces	18,700 (12.7%)	700 (0.14%)	0.03	300 (0.19%)	0.02	3,200 (0.58%)	0.17
70 μg Hb/g faeces	25,100 (17.0%)	900 (0.18%)	0.03	400 (0.26%)	0.02	4,400 (0.80%)	0.18
*Skipping screening ages*							
55-year-olds	9,200 (6.2%)	200 (0.04%)	0.02	100 (0.08%)	0.02	2,700 (0.48%)	0.29
63-year-olds	7,400 (5.0%)	200 (0.05%)	0.03	200 (0.10%)	0.02	2,300 (0.41%)	0.31
63- and 65-year-olds	16,100 (10.9%)	600 (0.12%)	0.03	400 (0.24%)	0.03	5,200 (0.93%)	0.32
*Extending the screening interval*							
28 months	12,600 (8.6%)	-200 (-0.04%)	-0.01	-200 (-0.12%)	-0.02	600 (0.11%)	0.05
30 months	19,000 (12.9%)	-200 (-0.03%)	-0.01	-300 (-0.19%)	-0.02	900 (0.16%)	0.05
32 months	18,500 (12.5%)	-200 (-0.05%)	-0.01	-500 (-0.27%)	-0.03	1,200 (0.21%)	0.06
34 months	18,000 (12.2%)	-200 (-0.05%)	-0.01	-600 (-0.35%)	-0.03	1,100 (0.20%)	0.06
36 months	16,200 (11.0%)	400 (0.08%)	0.02	-100 (-0.08%)	-0.01	5,300 (0.95%)	0.32

Abbreviations: CRC, colorectal cancer; LYs, Life years; μg Hb/g, microgram Haemoglobin per gram. Note that reductions and increases are compared to the comparator strategy, in which we simulated the Dutch CRC screening program including the 3-month disruption from April 2020 and after the disruption individuals’ missed invitations were caught up in the next 3 months.

### Skipping screening ages

Skipping just a single screening age would result in a reduction in colonoscopy demand of 9,200 (6.2%) for 55-year-old individuals and 7,400 (5.0%) for 63-year-old individuals. Skipping two screening ages (i.e. 63-year-old and 65-year-old individuals) would result in a reduction of 16,100 colonoscopies (10.8%) cumulatively over 2020, 2021 and 2022. Between 2020 and 2050, 600 additional CRC-related deaths (0.12%) were estimated in case of skipping two screening ages (63- and 65-year-olds). Skipping a single screening age would result in approximately 2,700 LYs lost (0.48%) and this would be doubled (5,200, 0.93%) when skipping two screening ages (63- and 65-year-olds).

### Extending screening interval

Extending the FIT screening interval only for individuals who were originally invited in the second half of 2020 and in the full year of 2021 would result in a reduction of 12,000 (8.6%) colonoscopies with an extension to 28 months, increasing up to 16,200 (11.0%) colonoscopies with a 36 months extension. Interestingly, the number of CRC-related deaths was estimated to be decreased as a result of the extension (-200 CRC-related deaths), because the extension led to individuals being screened at older ages. Extending the screening interval up to 34 months would result in the lowest increases in LYs lost compared to the other scenarios: 600 (0.11%) to 1,200 (0.21%) LYs were lost between 2020 and 2050. When extending the screening interval to 36 months, the number of LYs lost was estimated to be substantially higher at 5,300 (0.95%).

### Efficiency measures

Overall, skipping screening ages would result in the highest increase in CRC-related deaths per colonoscopy not performed (0.03 for skipping 63- and 65-year-olds) (Table 1). Increasing the FIT cut-off was the second-best option to reduce colonoscopy demand with lowest impact on the long-term CRC-related mortality. Extending the interval estimated the lowest decrease in CRC-related deaths per colonoscopy not performed (0.01 fewer CRC deaths per colonoscopy not performed). Moreover, extending the screening interval outperformed the strategies with skipping screening ages and increasing the cut-off. Even though extending the screening interval to 36 months resulted in the most estimated LYs lost in absolute terms, this strategy would result in the lowest increases in LYs lost per colonoscopy not performed (ranging from 0.05 to 0.32).

Extending the screening interval resulted in an estimated decrease in stage I, stage II and stage III in 2020 up to 750 cases, while estimated stage IV CRC cases remained similar between 2020 and 2023 compared to the comparator strategy (S4 Fig in S1 File). The decrease in diagnosed CRCs for each stage in 2020 is estimated to be caught up in the subsequent calendar years.

### Sensitivity analyses

Although assuming a 10 percentage point lower participation rate increased the estimated impact on both short-term outcomes (i.e. the colonoscopy demand) and long-term outcomes (i.e. CRC-related deaths and LYs lost), this assumption did not impact the best strategy to anticipate on restricted colonoscopy capacity (S2 Table in S1 File). If extending the screening interval would result in a cancellation of the last screening invitation for individuals aged >75 years, so 10 invitations in an individuals’ life-time rather than 11 invitations, an increase of up to 2,200 (1.29%) CRC-related deaths and 18,100 (3.26%) LYs lost was estimated between 2020 and 2050 (S3 Table in S1 File). Extending the screening interval was not the best strategy anymore. Extending the screening interval during a colonoscopy restriction period of 24 months was still the best strategy to reduce colonoscopy demand and minimize the impact on CRC incidence, CRC-related deaths and LYs lost (S4 Table in S1 File).

## Discussion

Our results predict that temporary restrictions in screening capacity have a modest impact on long-term outcomes, such as CRC incidence, CRC-related mortality and LYs lost. To anticipate on restrictions in colonoscopy capacity, temporarily extending the FIT screening interval to 34 months resulted in a high reduction in colonoscopy capacity in the short-term, whereas this strategy resulted in considerably fewer CRC diagnoses and LYs lost in the long-term compared to increasing the FIT cut-off value or skipping a screening age. If an additional reduction in colonoscopy demand would be required, increasing the FIT cut-off value in addition to extending the screening interval would be the most efficient option.

Interestingly, extending the screening interval estimated a decrease in CRC incidence and mortality from 2020–2050 compared to the comparator strategy of immediate catch-up. Obviously, the extension of the interval initially would result in an increase in CRC cases and CRC-related deaths, because a small proportion of advanced adenomas and early-stage CRCs that would normally be detected after the original screening interval, will now have progressed to (more advanced) CRC with the longer interval. However, because of the one-time extension of the interval, individuals are screened at a slightly older age in this strategy. For example, an individual whose interval has been extended to 30 months at age 71, will now be screened at ages 71.5, 73.5, and 75.5 instead of 71, 73 and 75. As CRC risk increases with age, the benefit of screening until a slightly older age due to a one-time longer interval in terms of CRC incidence and CRC-related mortality, is slightly larger than the loss in benefit from the slightly longer interval at younger ages. However, because life-expectancy is shorter at older ages, life-years gained associated with preventing cancers and deaths at these older ages are smaller than with cases and deaths from the standard screening interval. Therefore, extending the screening interval would still result in LYs lost compared to the comparator strategy, albeit the lowest increase in LYs lost per colonoscopy not performed (from 0.05 to 0.32 depending on the length of extension) of all scenarios considered. Also, our sensitivity analysis showed the importance to keep the total number of invitations as originally planned (i.e. to invite individuals for their last screening even if their screening age has exceeded the stopping age of the program).

During a pandemic, participation in FIT screening could be reduced due to fear of becoming infected and/or fear of adding pressure to the health care system. Lower participation reduces the effectiveness of the program and therefore results in more excess CRC diagnoses and ultimately in CRC-related deaths. Our sensitivity analyses showed that in case of decreased participation in FIT screening, extending the screening interval would still be the best strategy to temporarily decrease colonoscopy demand and minimize the long-term impact.

To our knowledge, this is the first modelling study on different strategies to temporarily reduce colonoscopy demand. Studies evaluating the impact of a delay between a positive FIT and follow up colonoscopy have been published. A systematic review showed that with delays of more than 9 months, incident CRC and advanced stage CRC increases with adjusted odd ratios of up to 1.75 [25]. Another study based published data created a model which predicted that the stage prevalence of advanced CRCs would increase from 26% with a delay of 0–3 months up to 29% with a delay of 7–12 months [26]. These extended intervals presented in previous studies, correspond with our extending interval of 34 months. Interestingly, we estimated also a serious impact on long-term outcomes at 36 months because at this extension the number of CRC cases prevented at older ages no longer outweighs the extra number of CRC cases occurring in the extended interval. Also, based on model estimates, extending the interval beyond 30 months would lead to a surplus in colonoscopy demand after 2021. Another modelling study showed that increasing the cut-off from 2 to 10 μg Hb/g faeces results in 18% lower colonoscopy demand [27]. Our study showed lower reductions possibly due to the higher FIT cut-off initially used: increasing the FIT cut-off from 47 to 55 μg Hb/g faeces results in 15,000 fewer colonoscopies, which is equal to a reduction of 10.2%. If an extension of 34 months would not reduce the colonoscopy demand sufficiently, additionally increasing the cut-off from 47 μg Hb/g faeces to 50 or 55 μg Hb/g faeces could be considered, since this would result in additional LYs lost (1,400 and 2,400, respectively). This is however still lower than to the LYs lost with an extension of 36 months (5,300 vs 2,000–3,300).

A key strength of this study is that it is the first study to assess different strategies (increasing the FIT cut-off, skipping screening ages and extending the screening interval) to temporarily reduce colonoscopy demand in an ongoing CRC screening program. Also, a well-established and validated microsimulation model was used for the predictions on colonoscopy demand and long-term screening outcomes such as CRC diagnoses, CRC-related deaths and LYs (lost).

Nevertheless, three limitations are noteworthy. First, the MISCAN-Colon model simulates the colonoscopy demand very straightforward as shown in Fig 1, resulting in lower and higher colonoscopy demand from 2022 to 2024. Especially in case of extending the screening interval to more than 30 months, the model estimated an increased demand in 2022 and 2024 and reduced demand in 2023. In practice, the colonoscopy demand could be spread out more by gradually returning to a screening interval of 24 months. The question remains whether an extension of more than 6 months is appropriate. This decision will most likely be driven by the necessity of reductions in colonoscopy demand in 2021. Second, the period during which the strategies need to be in effect is unknown, but since missed individuals needed to be caught up in the second half of 2020 and because of the large-scale roll-out of COVID-19 vaccination programs, one-and-a half year seemed a reasonable time frame. A sensitivity analysis evaluating restricted capacity for a period of 24 months after restart of the program resulted in similar conclusions with respect to the optimal mitigation strategy. Last, personalized screening using risk-stratification is becoming an important factor in improving the effectiveness of screening [28–30]. It could be used to prioritize individuals based on risk of CRC and therefore reduce the colonoscopy demand. However, we have not applied this in our study since personalized screening is quite complicated to implement in the short-term and the aim of this study was to assess the best strategy in the uniform screening program. Van Wifferen et al. evaluated prioritisation strategies by adjusting the FIT positivity cut-off by sex to mitigate the impact of the COVID-19 pandemic and found no benefit of this sex-based approach compared to uniform approaches [31].

In this paper, we focussed on reductions in colonoscopies directly associated with the screening program. Reductions in colonoscopy capacity could also be managed by changing the policy for diagnostic colonoscopies for symptom evaluation or surveillance colonoscopies. In England, for example, a low FIT positivity cut-off has been used for triage of patients with symptoms for colonoscopy during the COVID-19 pandemic, and lengthening of surveillance intervals for adenoma patients was already advocated even before the COVID-19 pandemic. However, these programs in the Netherlands are managed by the hospitals or even by medical doctors themselves. As such these colonoscopies cannot be influenced by the screening organization and they were therefore out of the scope of this study. We also did not consider alternative tests for triage of positive FIT, such as CT colonography or capsule endoscopy. Both are promising alternatives to colonoscopy, with capsule endoscopy having the additional advantage that it does not require hospital capacity [32]. However, given the high yield of colonoscopy after positive FIT, the benefit of triage is likely minimal.

Besides estimates for the colonoscopy capacity, other additional factors such as management, chemotherapies and need for surgeries are important to be estimated. Due to a delay in CRC screening, it can be expected that a stage shift would occur and therefore changes in cancer treatment. In the model, it is estimated that extending the screening interval up to 34 months results in a decrease in CRC incidence for stage I, II and III in 2020. This decrease is estimated to be caught up during the subsequent calendar years. Stage IV CRCs remained similar during 2020 and 2023. Earlier literature shows this drop in CRC incidence in the Netherlands in 2020 and to be caught later [33]. Both the model estimates and the observed data shows that cancer treatment is delayed rather than shifted to a different treatment option due to a CRC stage shift.

This study showed that temporarily extending the screening interval is a potential strategy to reduce colonoscopy demand, resulting in a relatively small impact on the long-term effect of screening. By considering three groups of strategies, we have created a guideline for healthcare providers and policy makers. Besides the application of this study during the COVID-19 pandemic, these results could be used to guide policy makers in future situations with temporarily restricted colonoscopy capacity within organized CRC screening programs such as new (waves of) pandemics, shortages of endoscopists and (natural) catastrophes. Earlier modelling studies have estimated the potential long-term impact of disruptions to CRC screening, showing the importance of continuing CRC screening [1] and with the strategies considered in this study CRC screening can be continued albeit at a lower colonoscopy capacity. Moreover, decision modelling using the MISCAN-Colon model has again provided relatively rapid insight in long-term impact on specific decisions that have to be made, as previously done at the start of the Dutch CRC screening program to lower colonoscopy demand [3].

## Conclusion

In case of temporarily restricted colonoscopy capacity, extending the screening interval to a maximum of 34 months in a FIT-based CRC screening program reduces colonoscopy demand, and therefore lowers the pressure on colonoscopy capacity while minimizing the impact on long-term CRC incidence, CRC-related mortality and LYs lost. If an even greater reduction in colonoscopy demand would be necessary, additionally increasing the FIT cut-off value could be considered.

## Supporting information

S1 File (DOCX)
