# Supplementary material for: Modelling optimal use of temporarily restricted colonoscopy capacity in a FIT-based CRC screening program: Application during the COVID-19 pandemic
Source: PLoS One. 2022 Jun 24;17(6):e0270223. doi: 10.1371/journal.pone.0270223 (PMC9231802; doi:10.1371/journal.pone.0270223)
Supplement: S1 File — (DOCX) [file pone.0270223.s001.docx]

Supporting information

Inhoud

[Tables 2](#_Toc98260432)

[Table S1 Test characteristics for FIT at different cut-offs and colonoscopy and FIT positivity- and detection rates observed in the Dutch national CRC screening program in 2014 and simulated by MISCAN-Colon positivity- and detection rates 2](#_Toc98260433)

[Table S2 The efficiency of strategies from the sensitivity analysis assuming 10 percentage point lower participation to FIT screening to reduce colonoscopy demand predicted by MISCAN-Colon. 3](#_Toc98260434)

[Table S3 The efficiency of strategies for extending the screening interval and cancelling last invitation to reduce colonoscopy demand predicted by MISCAN-Colon. 4](#_Toc98260435)

[Figures 6](#_Toc98260436)

[Figure S1 Modelled comparator strategy in case of restrictions on colonoscopy capacity in CRC screening 6](#_Toc98260437)

[Figure S2 Modelled strategies in which FIT positivity cut-off was increased or screening ages were skipped in case of restrictions on colonoscopy capacity in CRC screening 7](#_Toc98260438)

[Figure S3 Modelled strategy with extended screening interval in case of restrictions on colonoscopy capacity in CRC screening 8](#_Toc98260439)

[Figure S4 Difference in CRC incidence by stage in case of extending the screening interval during 18 months after restart of the screening program. 9](#_Toc98260440)

[References 10](#_Toc98260441)

# Tables

## Table S1 Test characteristics for FIT at different cut-offs and colonoscopy and FIT positivity- and detection rates observed in the Dutch national CRC screening program in 2014 and simulated by MISCAN-Colon positivity- and detection rates

|  | FIT47 | FIT50 | FIT55 | FIT60 | FIT70 | Colonoscopy |
| --- | --- | --- | --- | --- | --- | --- |
| Sensitivity |  |  |  |  |  |  |
| Small adenomas (≤ 5 mm) | 0% | 0% | 0% | 0% | 0% | 75% |
| Medium adenomas (6-9 mm) | 4.5% | 4.3% | 3.8% | 3.4% | 2.8% | 85.0% |
| Large adenomas (≥10 mm) | 30.7% | 29.9% | 28.7% | 27.6% | 25.6% | 85.0% |
| CRC early preclinical ^a^ | 32.4% | 31.4% | 30.7% | 30.0% | 28.8% | 95.0% |
| CRC late preclinical | 68.1% | 68.0% | 67.3% | 66.6% | 65.3% | 95.0% |
| Specificity | 97.7% | 98.0% | 98.1% | 98.2% | 98.4% | 100.0% |
|  |  |  |  |  |  |  |
| Positivity rate |  |  |  |  |  |  |
| Observed ^b^ | 0.0640 | 0.0610 | 0.0580 | 0.0550 | 0.0510 |  |
| Simulated | 0.0635 | 0.0615 | 0.0583 | 0.0555 | 0.0506 |  |
| Detection rate non-advanced adenomas |  |  |  |  |  |  |
| Observed ^b^ | 0.0115 | 0.0109 | 0.0100 | 0.0093 | 0.0080 |  |
| Simulated | 0.0115 | 0.0189 | 0.0100 | 0.0093 | 0.0080 |  |
| Detection rate advanced adenomas |  |  |  |  |  |  |
| Observed ^b^ | 0.0314 | 0.0308 | 0.0295 | 0.0284 | 0.0263 |  |
| Simulated | 0.0315 | 0.0376 | 0.0295 | 0.0284 | 0.0263 |  |
| Detection rate CRC |  |  |  |  |  |  |
| Observed ^b^ | 0.0060 | 0.0059 | 0.0058 | 0.0057 | 0.0055 |  |
| Simulated | 0.0060 | 0.0059 | 0.0058 | 0.0057 | 0.0055 |  |

Abbreviations: FIT47, faecal immunochemical test using a cut-off of 47 µg Hb/g faeces; FIT50, faecal immunochemical test using a cut-off of 50 µg Hb/g faeces; FIT55, faecal immunochemical test using a cut-off of 55 µg Hb/g faeces; FIT60, faecal immunochemical test using a cut-off of 60 µg Hb/g faeces; FIT70, faecal immunochemical test using a cut-off of 70 µg Hb/g faeces; CRC, colorectal cancer.

1. It was assumed that the probability a CRC bleeds and thus the sensitivity of a FIT for CRC depends on the time until clinical diagnosis.[1]
2. The observed detection rates were corrected for lack of adherence with colonoscopy to allow unbiased comparison with estimated detection rates. This is established by multiplying the observed positivity rate with the positive predictive value.

## Table S2 The efficiency of strategies from the sensitivity analysis assuming 10 percentage point lower participation to FIT screening to reduce colonoscopy demand predicted by MISCAN-Colon.

|  | Reduction in colonoscopy demand in 2020, 2021 and 2022 (%) | Excess CRC incidence  (2020-2050, %) | Increase in CRC incidence per colonoscopy not performed | Excess CRC deaths  (2020-2050, %) | Increase in CRC deaths per colonoscopy not performed | Excess LYs lost  (2020-2050, %) | Increase in CRC LYs lost per colonoscopy not performed |
| --- | --- | --- | --- | --- | --- | --- | --- |
| *Increasing the FIT cut-off value* | | | |  |  |  |  |
| 50 µg Hb/g faeces | 19,300 (13.1%) | 600 (0.12%) | 0.03 | 400 (0.21%) | 0.02 | 4,300 (0.76%) | 0.22 |
| 55 µg Hb/g faeces | 23,000 (15.6%) | 700 (0.15%) | 0.03 | 400 (0.26%) | 0.02 | 4,700 (0.90%) | 0.20 |
| 60 µg Hb/g faeces | 26,300 (17.8%) | 800 (0.18%) | 0.03 | 500 (0.30%) | 0.02 | 5,500 (1.05%) | 0.21 |
| 70 µg Hb/g faeces | 31,800 (21.5%) | 1,000 (0.22%) | 0.03 | 600 (0.36%) | 0.02 | 6,900 (1.26%) | 0.22 |
| *Skipping screening ages* | |  |  |  |  |  |  |
| 55-year-olds | 18,100 (12.3%) | 500 (0.11%) | 0.03 | 400 (0.22%) | 0.02 | 5,100 (0.92%) | 0.28 |
| 63-year-olds | 16,400 (11.1%) | 500 (0.11%) | 0.03 | 400 (0.24%) | 0.02 | 5,000 (0.96%) | 0.30 |
| 63- and 65-year-olds | 24,100 (16.3%) | 700 (0.16%) | 0.03 | 600 (0.34%) | 0.03 | 7,200 (1.35%) | 0.30 |
| *Extending the screening interval* | | | |  |  |  |  |
| 28 months | 23,400 (15.9%) | 100 (0.02%) | 0.00 | 0 (0.00%) | 0.00 | 3,700 (0.73%) | 0.16 |
| 30 months | 30,300 (20.5%) | 100 (0.02%) | 0.00 | -100 (-0.05%) | 0.00 | 3,800 (0.67%) | 0.13 |
| 32 months | 29,800 (20.2%) | 100 (0.01%) | 0.00 | -200 (-0.12%) | -0.01 | 4,000 (0.76%) | 0.13 |
| 34 months | 29,300 (19.9%) | 0 (0.00%) | 0.00 | -400 (-0.22%) | -0.01 | 3,900 (0.78%) | 0.13 |
| 36 months | 27,800 (18.8%) | 700 (0.14%) | 0.02 | 100 (0.06%) | 0.00 | 8,100 (1.52%) | 0.29 |

Abbreviations: CRC, colorectal cancer; LYs, life years; µg Hb/g, microgram Haemoglobin per gram. Note that reductions and increases are compared to the comparator strategy, in which we simulated the Dutch CRC screening program including the 3-month disruption from April 2020 and after the disruption individuals’ missed invitations were caught up in the next 3 months.

## Table S3 The efficiency of strategies for extending the screening interval and cancelling last invitation to reduce colonoscopy demand predicted by MISCAN-Colon.

|  | Reduction in colonoscopy demand in 2020, 2021 and 2022 (%) | Excess CRC incidence  (2020-2050, %) | Increase in CRC incidence per colonoscopy not performed | Excess CRC deaths  (2020-2050, %) | Increase in CRC deaths per colonoscopy not performed | Excess LYs lost  (2020-2050, %) | Increase in CRC LYs lost per colonoscopy not performed |
| --- | --- | --- | --- | --- | --- | --- | --- |
| *Cancelling last invitation* | | | |  |  |  |  |
| 28 months | 14,000 (9.5%) | 1,500 (0.33%) | 0.11 | 2,200 (1.28%) | 0.16 | 15,000 (2.70%) | 1.07 |
| 30 months | 19,800 (13.4%) | 1,400 (0.31%) | 0.07 | 2,000 (1.18%) | 0.10 | 14,600 (2.63%) | 0.74 |
| 32 months | 19,300 (13.1%) | 1,400 (0.30%) | 0.07 | 1,900 (1.09%) | 0.10 | 14,700 (2.65%) | 0.76 |
| 34 months | 18,800 (12.7%) | 1,300 (0.29%) | 0.07 | 1,800 (1.01%) | 0.09 | 14,500 (2.61%) | 0.77 |
| 36 months | 17,000 (11.5%) | 1,900 (0.40%) | 0.11 | 2,100 (1.20%) | 0.12 | 17,800 (3.21%) | 1.04 |

Abbreviations: CRC, colorectal cancer; LYs, Life years. Note that reductions and increases are compared to the comparator strategy, in which we simulated the Dutch CRC screening program including the 3-month disruption from April 2020 and after the disruption individuals’ missed invitations were caught up in the next 3 months.

Table S4 The efficiency of strategies from the sensitivity analysis assuming a restriction period of 24 months to reduce colonoscopy demand predicted by MISCAN-Colon.

|  | Reduction in colonoscopy demand in 2020, 2021 and 2022 (%) | Excess CRC incidence  (2020-2050, %) | Increase in CRC incidence per colonoscopy not performed | Excess CRC deaths  (2020-2050, %) | Increase in CRC deaths per colonoscopy not performed | Excess LYs lost  (2020-2050, %) | Increase in CRC LYs lost per colonoscopy not performed |
| --- | --- | --- | --- | --- | --- | --- | --- |
| *Increasing the FIT cut-off value* | | | |  |  |  |  |
| 50 µg Hb/g faeces | 11,000 (7.5%) | 500 (0.10%) | 0.04 | 200 (0.12%) | 0.02 | 2,100 (0.38%) | 0.19 |
| 55 µg Hb/g faeces | 15,400 (10.5%) | 600 (0.14%) | 0.04 | 300 (0.18%) | 0.02 | 2,900 (0.52%) | 0.19 |
| 60 µg Hb/g faeces | 19,300 (13.1%) | 800 (0.17%) | 0.04 | 400 (0.24%) | 0.02 | 4,000 (0.72%) | 0.21 |
| 70 µg Hb/g faeces | 25,800 (17.5%) | 1,100 (0.25%) | 0.04 | 600 (0.33%) | 0.02 | 5,500 (0.99%) | 0.21 |
| *Skipping screening ages* | |  |  |  |  |  |  |
| 55-year-olds | 12,200 (8.2%) | 300 (0.06%) | 0.02 | 200 (0.12%) | 0.02 | 3,400 (0.62%) | 0.28 |
| 63-year-olds | 7,400 (5.0%) | 200 (0.05%) | 0.03 | 200 (0.10%) | 0.02 | 2,300 (0.41%) | 0.31 |
| 63- and 65-year-olds | 16,100 (10.9%) | 600 (0.12%) | 0.03 | 400 (0.24%) | 0.03 | 5,200 (0.93%) | 0.32 |
| *Extending the screening interval* | | | |  |  |  |  |
| 28 months | 12,400 (8.4%) | -200 (-0.03%) | -0.01 | -200 (-0.11%) | -0.02 | 1,300 (0.24%) | 0.11 |
| 30 months | 18,700 (12.7%) | -300 (-0.06%) | -0.01 | -400 (-0.24%) | -0.02 | 1,100 (0.19%) | 0.06 |
| 32 months | 18,500 (12.5%) | -200 (-0.05%) | -0.01 | -500 (-0.27%) | -0.03 | 1,200 (0.21%) | 0.06 |
| 34 months | 23,100 (15.6%) | -200 (-0.05%) | -0.01 | -700 (-0.39%) | -0.03 | 1,600 (0.29%) | 0.07 |
| 36 months | 29,600 (20.0%) | 500 (0.11%) | 0.02 | -200 (0.09%) | -0.01 | 6,500 (1.17%) | 0.22 |

Abbreviations: CRC, colorectal cancer; LYs, life years; µg Hb/g, microgram Haemoglobin per gram. Note that reductions and increases are compared to the comparator strategy, in which we simulated the Dutch CRC screening program including the 3-month disruption from April 2020 and after the disruption individuals’ missed invitations were caught up in the next 3 months.

# Figures


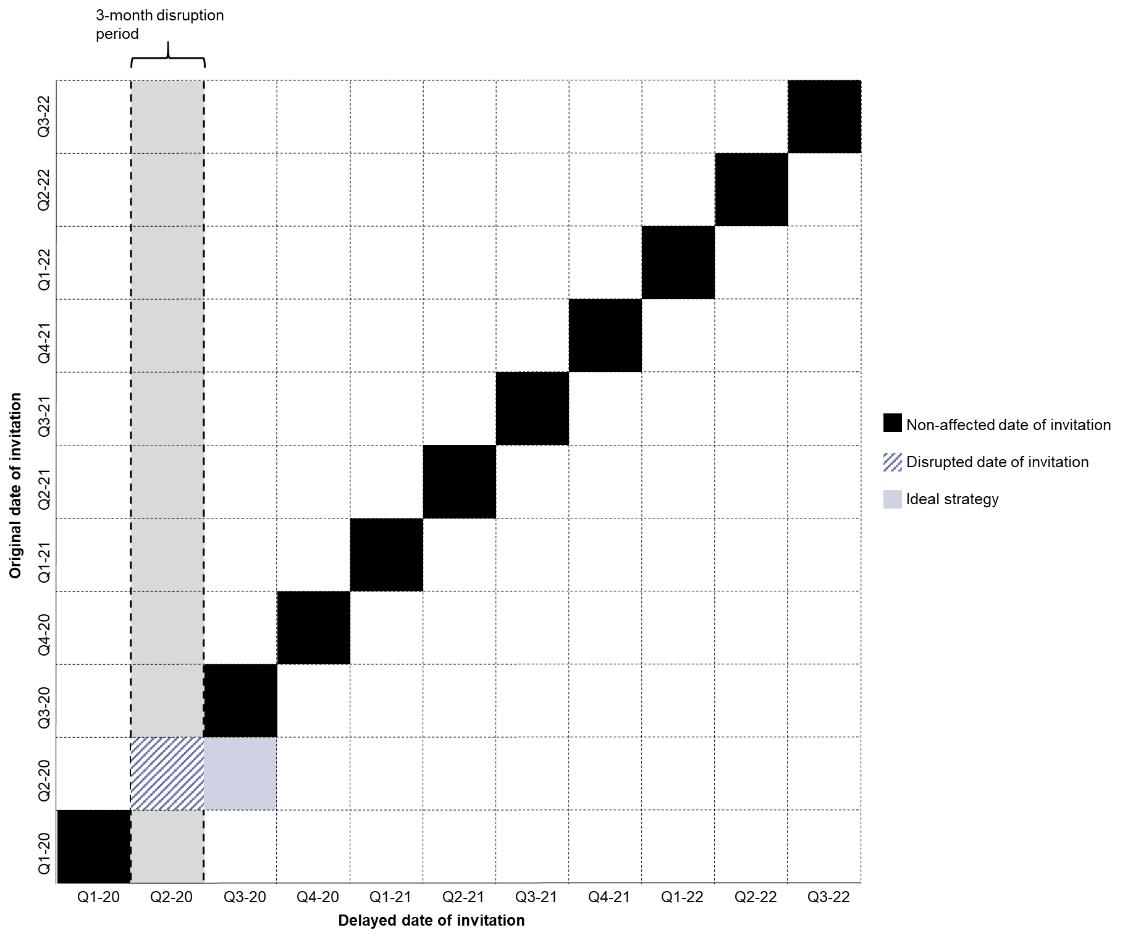


## Figure S1 Modelled comparator strategy in case of restrictions on colonoscopy capacity in CRC screening

Abbreviations: CRC, colorectal cancer; Q1, first quarter in a calendar year (January-March); Q2, second quarter in a calendar year (April-June); Q3, third quarter in calendar year (July-September); Q4, fourth quarter in calendar year (October-December).


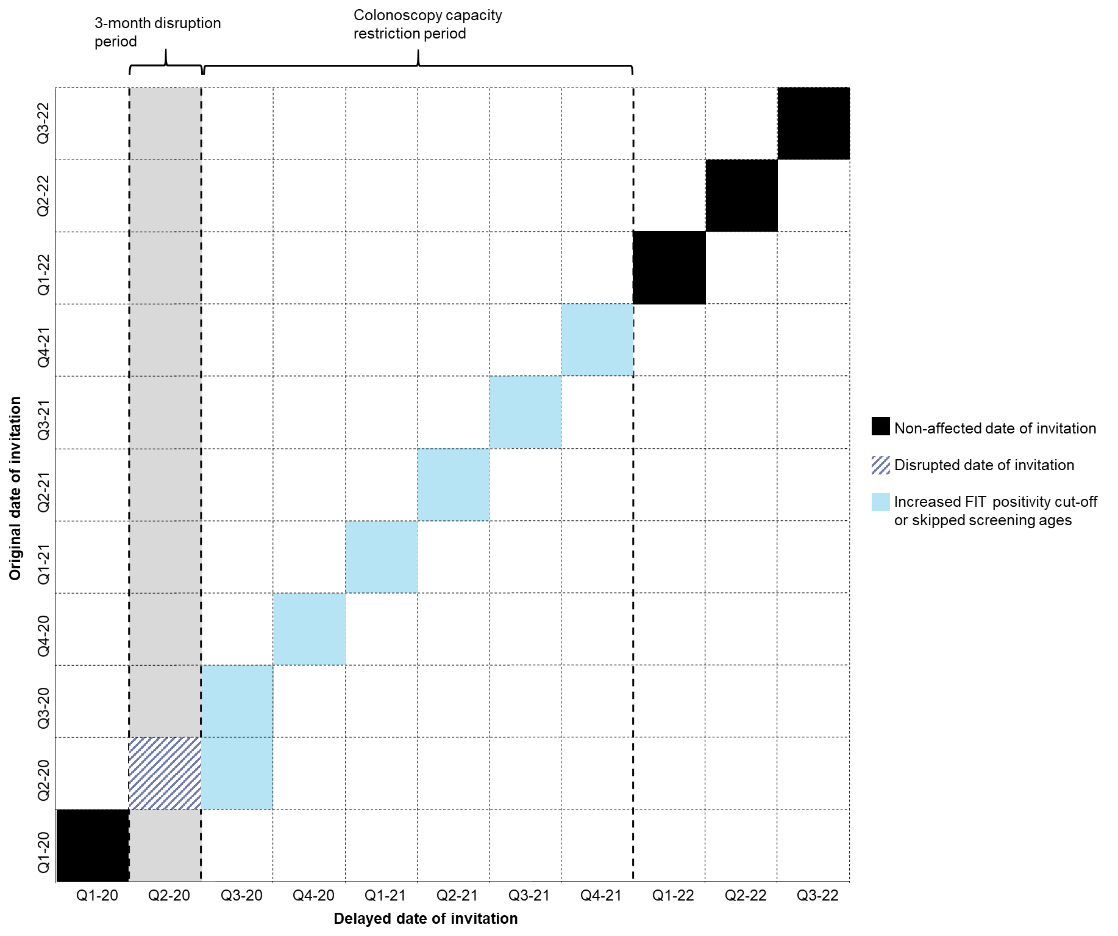


## Figure S2 Modelled strategies in which FIT positivity cut-off was increased or screening ages were skipped in case of restrictions on colonoscopy capacity in CRC screening

Abbreviations: FIT, faecal immunochemical test; CRC, colorectal cancer; Q1, first quarter in a calendar year (January-March); Q2, second quarter in a calendar year (April-June); Q3, third quarter in calendar year (July-September); Q4, fourth quarter in calendar year (October-December).


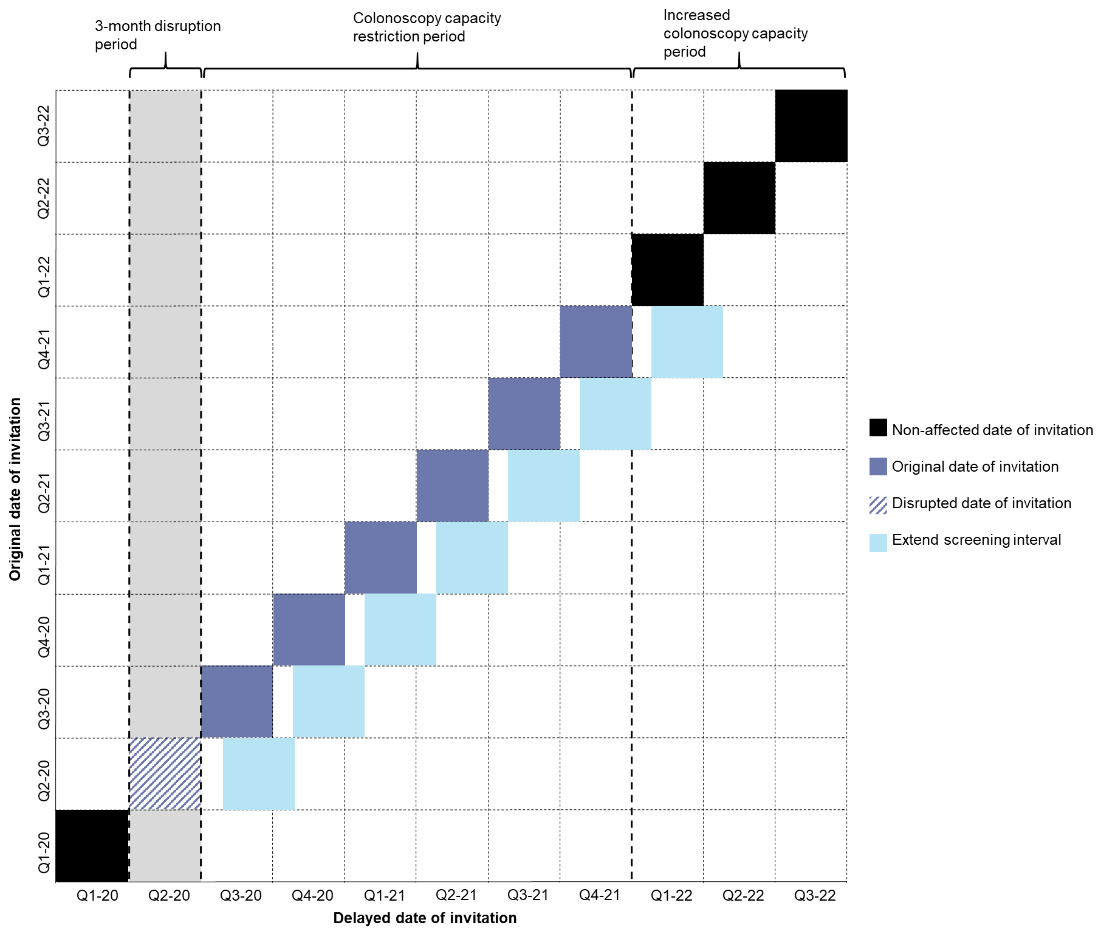


## Figure S3 Modelled strategy with extended screening interval in case of restrictions on colonoscopy capacity in CRC screening

Abbreviations: CRC, colorectal cancer; Q1, first quarter in a calendar year (January-March); Q2, second quarter in a calendar year (April-June); Q3, third quarter in calendar year (July-September); Q4, fourth quarter in calendar year (October-December).


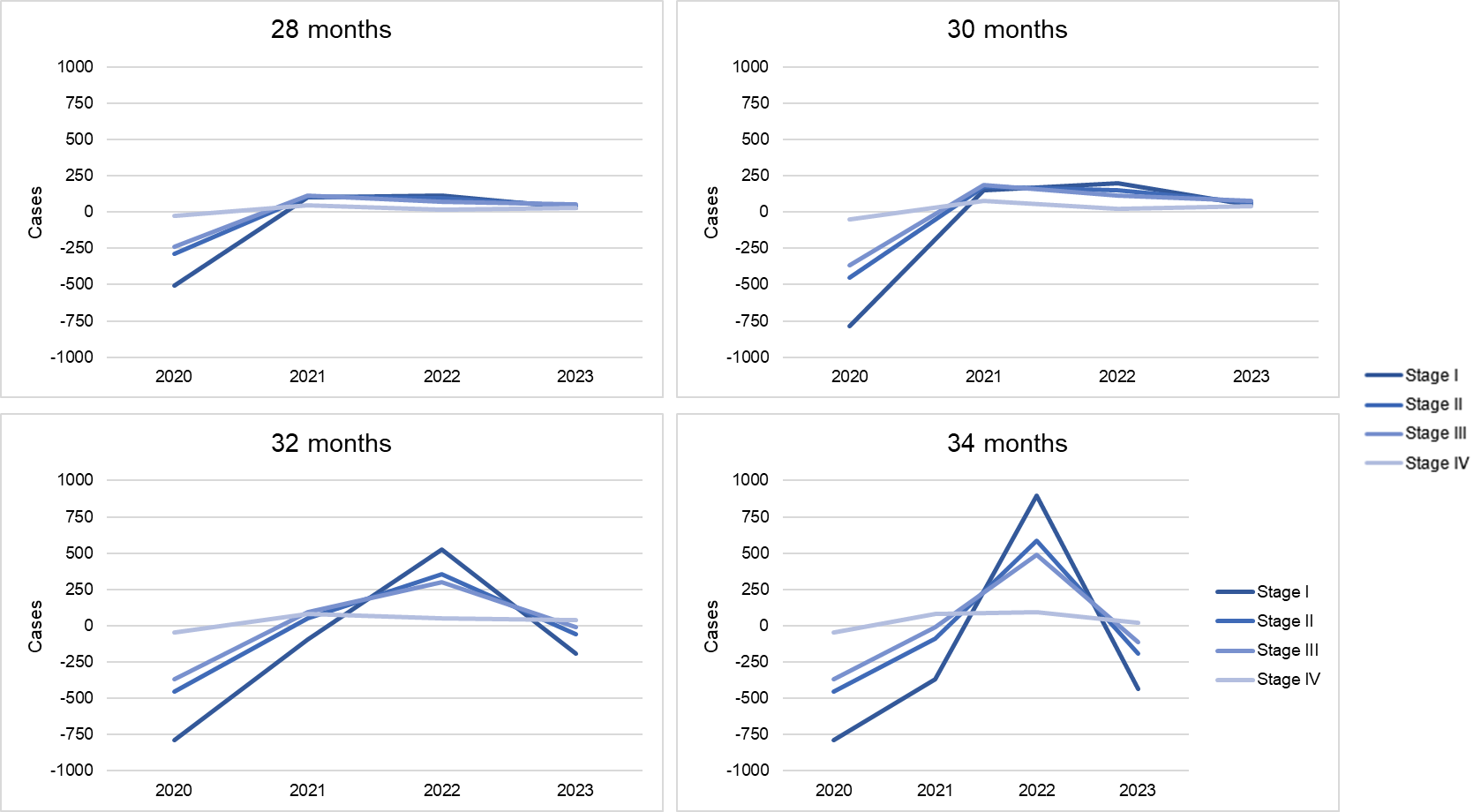


## Figure S4 Difference in CRC incidence by stage in case of extending the screening interval during 18 months after restart of the screening program.

# References

1. Lansdorp-Vogelaar, I., et al., *A novel hypothesis on the sensitivity of the fecal occult blood test: Results of a joint analysis of 3 randomized controlled trials.* Cancer, 2009. **115**(11): p. 2410-9.
